# Identification and Validation of Reliable Reference Genes for Gene Expression Studies in *Koelreuteria paniculata*

**DOI:** 10.3390/genes13050714

**Published:** 2022-04-19

**Authors:** Kai Gao, Wasif Ullah Khan, Juan Li, Sai Huang, Xiong Yang, Ting Guo, Bin Guo, Ruqian Wu, Xinmin An

**Affiliations:** 1 Beijing Advanced Innovation Center for Tree Breeding by Molecular Design, National Engineering Research Center of Tree breeding and Ecological Restoration, College of Biological Sciences and Technology, Beijing Forestry University, Beijing 100083, China; gaokai@caf.ac.cn (K.G.); Wasifullah@bjfu.edu.cn (W.U.K.); lj859851939@bjfu.edu.cn (J.L.); HUANGsai@bjfu.edu.cn (S.H.); xiongyang@bjfu.edu.cn (X.Y.); guoting2020@bjfu.edu.cn (T.G.); guobin1986@bjfu.edu.cn (B.G.); RuqianWu1205@bjfu.edu.cn (R.W.); 2 Research Institute of Subtropical Forestry, Chinese Academy of Forestry, Hangzhou 311400, China; 3 Shanxi Academy of Forestry and Grassland Sciences, Taiyuan 030012, China

**Keywords:** *Koelreuteria paniculata*, RT-qPCR, reference genes, plant tissues, embryo developmental stages

## Abstract

RT-qPCR is considered a rapid and reliable technique for analyzing gene expression. This technique is commonly used to analyze the expression of various genes at diverse transcriptional levels in different samples. However, few studies have characterized ornamental *Koelreuteria* species for reliable reference genes. In this study, eight reference genes were evaluated as controls in RT-qPCR with SYBR green to quantify gene expression in different *Koelreuteria paniculata* samples. All selected reference genes showed a broad range of C_t_ values in all samples, which was supportive of their variable expression. Our results showed significant variation in the stable expression of *K. paniculata* genes. Sample data, analyzed using geNorm, NormFinder, and BestKeeper, showed that phospholipase (*PLA2*) and β-actin (*ACT*) were the most suitable and statistically reliable reference genes, whereas ribosomal protein L13 (*RPL13*) and elongation factor 1-α (*EF1α*) were less stable and unsuitable for use as internal controls. To compare gene expression levels, two or more reference genes should be used for data normalization. Thus, the stability and expression of both *PLA2* and *ACT* were believed to provide better normalization and quantification of the transcript levels for gene expression studies in *K. paniculata*.

## 1. Introduction

Reverse transcriptase quantitative real-time polymerase chain reaction (RT-qPCR) is a sensitive, specific, and reproducible technique used to examine the mRNA transcription levels of a specific gene of interest [1,2,3,4]. It can be used to compare RNAs that show significant variation. RT-qPCR amplification products are often detected using two approaches; one approach is based on the relative quantification method of housekeeping genes (HKGs) as reference genes, while the other method is absolute quantification, in which the expression level of DNA is determined using a standard curve [5].

Relative quantification is one of the simplest and most effective techniques used to detect changes in gene expression levels. To avoid misinterpreting data and reporting inaccurate experimental results, most studies use a reference gene. Data generated through RT-qPCR are normalized by simultaneously expressing and detecting the target gene expression levels and the reference gene in the same sample [4,6,7,8].

Internal reference or control genes under various conditions—such as in different developmental stages or tissue types—should be expressed at a constant level and should not be affected by experimental parameters [9]. In recent studies, the stability of several housekeeping genes, including 18S ribosomal RNA (*18SrRNA*), *ACT*, and glyceraldehydes-3-phosphate dehydrogenase (*GAPDH*), was questioned based on their diverse behaviors under different experimental conditions [10,11,12,13,14]. Moreover, analyses of second-generation sequencing data from various species and their internal reference gene expression have shown species-specific expression and unique expression in different tissues. Additionally, expression of these reference genes is affected by environmental factors, such as water, light, and temperature [5,15,16,17,18,19]. Therefore, at least two reference genes should be expressed at constant levels under various conditions to normalize the relative quantitative results.

Internal reference genes should be validated in specific experimental models. Previous studies have identified internal reference genes in several species, including wheat [20], tomato [21], potato [14], flax [5], eucalyptus [22], *Arabidopsis* [23], rice [24], sesame [25], and *Papaya* [26]. However, to the best of our knowledge, there have been no reports of RT-qPCR studies on the differential expression of reference genes in *K. paniculata*.

In this study, eight reference genes were selected to evaluate the stability of normalization in *Koelreuteria* gene expression. We established a regeneration system for *K. paniculata* and obtained a sterile tissue culture, allowing us to perform this study in vitro. We also selected the interested genes for validation by referring to the transcriptome data. Statistical methods incorporated in geNorm [27], NormFinder [28], and BestKeeper [29] were used.

## 2. Materials and Methods

### 2.1. Plant Materials

Immature seeds from *K. paniculata* were collected from October to November and planted at Beijing Forestry University (Beijing, China). Seeds were aseptically cultured to obtain sterile seedlings, after which embryogenic callus was induced to obtain plants from somatic embryogenesis and the embryos were collected. Embryo development was divided into three phases: early embryogenesis, late embryogenesis, and mature cotyledon embryogenesis. Fully mature cotyledon embryos developed into whole plants in rooting medium, and samples were collected [30]. The roots, stems, and leaves of the sterile plants were collected after 3 months of growth. Tissue culture materials were cultured at 25 ± 2 °C. Light culture materials were placed in a 16/8 h photoperiod environment with a light intensity of 40 μmol m^−2^ s^−1^. Additionally, leaves of two different *K. paniculata* (wild type and yellow leaf mutant) were obtained from adult trees in a nursery at Xiangyuan (Changzhi, China), and the leaves were used for further expression validation analyses. All samples were immediately frozen in liquid nitrogen and stored at −80 °C until further use.

### 2.2. Total RNA Isolation and cDNA Synthesis

Total RNA was extracted using the SV Total RNA Isolation System (Promega, Madison, WI, USA). RNA was quantified based on absorbance at OD260 using the NanoDrop-2000 spectrophotometer (Thermo Scientific, Waltham, MA, USA). The absorbance ratios at OD260/280 and OD260/230 were used to assess the purity of all RNA samples. RNA samples were used for further analysis with an OD260/280 ratio (protein contamination) between 1.8–2.0 and OD260/230 (organic pollutant) higher than 2.0. RNA integrity was verified based on 2% agar gel electrophoresis and ethidium bromide staining. One microgram of RNA was reverse-transcribed using the SYBR PrimeScript RT-PCR kit II (TaKaRa, Dalian, China) for first-strand cDNA synthesis according to the manufacturer’s instructions. Before transcription, RNA and primers were mixed and incubated at 70 °C for 5 min, followed by immediate cooling on ice. First-strand cDNA synthesis was initiated after adding the transcription mixture at 37 °C, and the reverse transcriptase reaction was performed for 15min. Finally, the PrimeScript Reverse Transcriptase was inactivated by heating the reaction mixture for 15 min at 75 °C. All cDNA samples were stored at −20 °C before being used as templates for RT-qPCR analysis.

### 2.3. Selection of Candidate K. paniculata Genes and Primer Design

Eight genes were selected to identify the most stably expressed reference genes in RT-qPCR studies. Genes included are considered typical internal reference genes commonly used for gene expression studies, such as *GAPDH*, *18S rRNA*, *ACT*, and others, based on previous reports [31,32,33]. As the complete *K. paniculata* genome was not available at the time of this study, we identified the gene identifiers and gene sequences of eight candidate genes from *K. paniculata* transcriptome sequencing results (unpublished). These genes included: *ACT*, *EF1α*, *GAPDH*, *RPL13*, *PLA2*, ubiquitin (*UBQ*), cyclophilin (*CYP2*), and RNA polymerase II (*RP II*) (Table 1). Before primer design, alignments were made with the DNA sequence of relevant orthologs in Arabidopsis, and the chosen candidate gene sequences were used to query the Arabidopsis database using BLAST to obtain a description of *K. paniculata* reference genes. Primers were designed with Primer Premier 5 with melting temperatures of 60 °C, for 22bp and about 50% GC content (Table 2). Each pair of primers was tested using endpoint PCR before RT-qPCR.

### 2.4. Quantitative Real-Time PCR Analysis

RT-qPCR was performed using a DNA Engine (MJ Research, New Haven, CT, USA) and the SYBR Premix Ex Taq polymerase (TaKaRa, Toyoto, Japan). The PCR reaction volume was 25 μL containing 2 μL of diluted cDNA, 12.5 μL 2 × SYBR Premix and 0.2 μM of each primer. The RT-qPCR programs were as follows: a pre-denaturation of 95 °C/2 min, followed by 40 cycles of 94 °C/15 s, 60 °C/20 s, and 72 °C/15 s. The amplification efficiency was calculated using the following formula: efficiency = 10^(−1/slope)^ − 1. Moreover, a melting curve analysis was performed at the end of program to determine the validity of experimental results. All reactions were performed in triplicate for technical and biological replicates, and each biological repetition was performed by mixing the tissue sample from three individuals.

### 2.5. Reference Gene Validation

RNA-seq data were obtained from two different *K. paniculata* leaves (wild type and yellow leaf mutant) using Illumina Hiseq platform. The yellow leaves mutant was originated from the wild type using grafting techniques under the same growth conditions; based on the RNA-seq data, individual genes had different expression levels across all tested samples. Therefore, we focused on the related genes in the chlorophyll metabolism pathway based on the leaf color of *K. paniculata*.

In total, 6 genes (2 upregulated, 2 downregulated, and 2 with no change in transcript abundance) were selected from the *K. paniculata* transcriptome sequencing results (unpublished data) for validation. Four candidate genes (*PLA2*, *ACT*, *RPL13* and *EF1α*) were selected as the reference gene for normalization using the 2^−ΔΔCt^ method. The sequences of the genes selected for validation and the primers used are summarized in Table S1.

### 2.6. Data Analysis

The expression levels of the tested reference genes were determined by calculating cycle threshold (C_t_) values. All amplification plots were analyzed with a threshold fluorescence value of 0.1 to obtain C_t_ values using the MJ Opticon Monitor analysis software version 3.1 (MJ Research). Results from the MJ Opticon Monitor analysis software were imported into Microsoft Excel and transformed to relative quantities using the comparative C_t_ method and specific efficiencies for each gene. The data were obtained and converted to correct input files, according to the requirements of the software, and analyzed using three different VBA applets: geNorm [27], NormFinder [28], and BestKeeper [29].

## 3. Results

RT-qPCR assays based on SYBR green detection were designed for eight genes (*ACT*, *EF1α*, *GAPDH*, *UBQ*, *CYP2*, *RPL13*, *PLA2*, and *RP II*; Table 1) in order to identify reliable genes with which to study *K. paniculata* gene expression. The specificity of the amplification was confirmed based on the presence of a single band of the expected size for each primer pair in agarose gels following electrophoresis and a single-peak melting curve analysis of the PCR products (Figures S1 and S2). The melting temperature of each gene after RT-qPCR amplification showed no primer dimer and could be used for subsequent RT-qPCR experiments. The effectiveness of PCR reactions varied from 1.79 for *CYP2* to 2.10 for *ACT*.

### 3.1. Expression Profiles of Reference Genes

**Figure 1:**
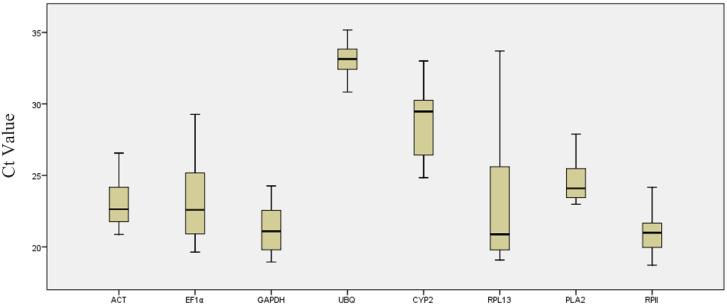
RT-qPCR C_t_ values for reference genes. Expression data are shown as C_t_ values for each reference gene in all *K. paniculata* samples. A line across the box depicts the median. The box indicates the 25th and 75th percentiles. Whiskers represent the maximum and minimum values.

C_t_ values of all candidate internal reference genes were obtained by analyzing data results from different samples. The results showed significant variation among the expression levels of HKGs across all samples (Figure 1). The C_t_ values for the eight studied genes varied from 18.72 to 35.17. However, most values ranged between 18 and 26.

Among the studied reference genes, *ACT*, *GAPGH*, *UBQ*, *PAL2,* and *RP II* showed lower (fewer than 6 cycles) gene expression, whereas *EF1α* and *RPL13* showed higher (more than 9 cycles) expression variations. The *RP II* encoding genes had the highest expression levels and reached the threshold after 18.72 cycles of amplification; however, the C_t_ average of all reference genes within the datasets was approximately 24.8 cycles. The least abundant transcripts were *CYP2* and *UBQ*, with C_t_ values of 28.79 and 33.17, respectively. The expression levels of HKGs differed under different conditions. The widespread expression of the eight tested reference genes confirmed that no single reference gene had a constant expression in different *K. paniculata* samples. Therefore, in order to analyze the experimental results, it was important to choose a reliable reference gene. 

### 3.2. GeNorm Analysis

**Figure 2:**
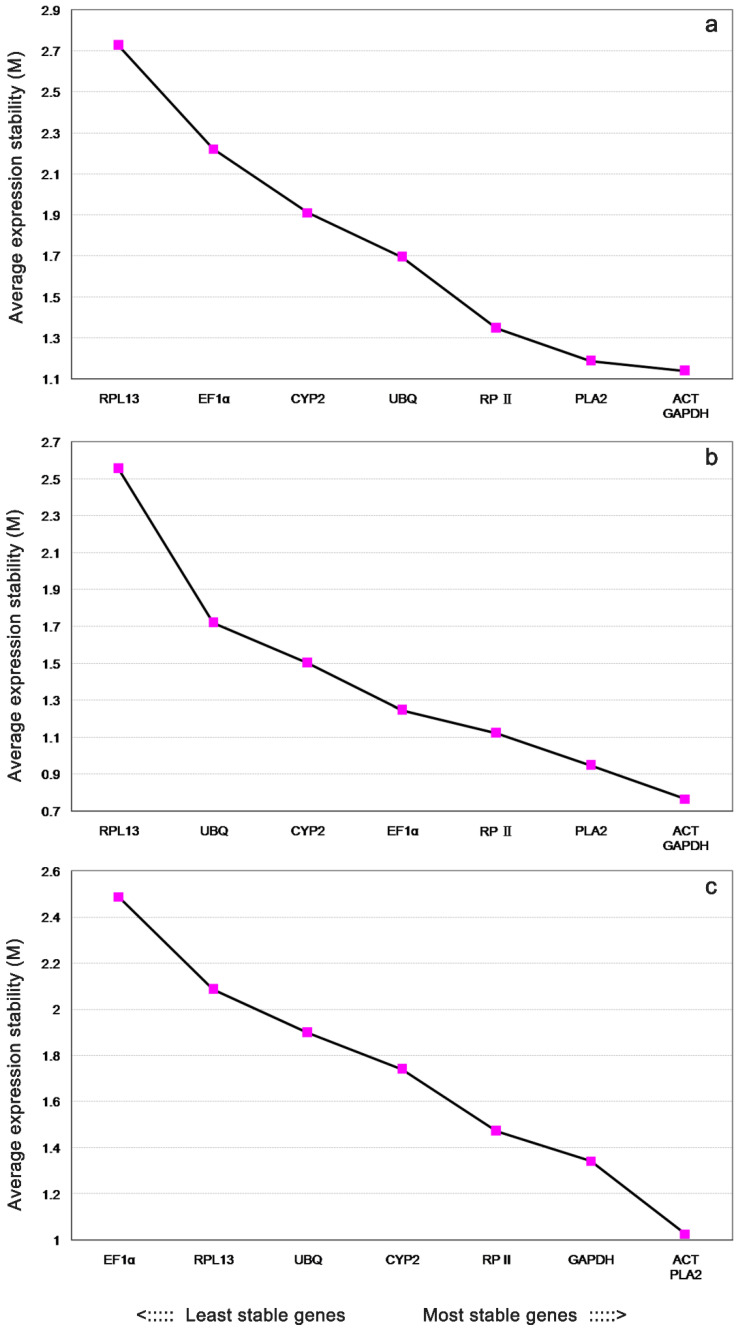
Gene expression stability and ranking of the eight reference genes as caculated by geNorm. Expression stability and ranking of the eight reference genes as caculated with geNorm in all samples (**a**), different vegetative tissues (**b**), and embryonic developmental stages (**c**). A lower average expression stability (M value) indicates more stable expression.

The statistical software geNorm was used to rank the gene expression stability of these eight reference genes. The M-value is the average degree of change in the expression level of a given gene relative to that of other HKGs. The expression M values of the eight reference genes are plotted in Figure 2. The reference gene, with a low M value, was marked as a highly stable expressed gene, whereas genes with high M values showed the least stable expression. In accordance with these principles, geNorm removed a candidate housekeeping gene with the lowest stability each time, gradually eliminating the less stable housekeeping candidate genes until the last two HKGs, with the highest stability, were selected as internal reference genes.

All samples were taken together, as shown in Figure 2a. The average expression stability values (M) of ACT and GAPDH were lowest, whereas that of RPL13 was highest, indicating that ACT and GAPDH had the most stable expression and RPL13 was expressed most variably. Similarly, in different tissue samples, ACT and GAPDH were the most stable genes, whereas RPL13 was the least stable (Figure 2b). Overall, ACT and PLA2 were the most stable genes, whereas EF1α, which had the highest M value, had the most variable expression during embryonic developmental stages (Figure 2c).

### 3.3. NormFinder Analysis

NormFinder is a Visual Basic application tool for Microsoft Excel that mainly uses the model method to identify the best genes among a set of candidate genes. The dataset that the program requires to import into the algorithm contains at least three candidate HKGs, and each gene is expressed in more than two samples. In this mathematical design, estimates of gene expression stability values include estimates of intra- and inter-group variation and individual analysis of sample-subgroup expression levels. More stable gene expression is indicated by a lower average expression stability value. Under our experimental conditions, candidate internal reference genes should have a stability value corresponding to a specific HKG that is small or even close to zero. In our study, six sample-subgroups were established for geNorm analysis. Moreover, expression data were combined into “plant tissues” (stems, roots, leaves) and “embryonic developmental stages” (early embryogenesis, late embryogenesis, and mature cotyledon embryogenesis) sample-subgroups. Meanwhile, all samples without subgroups were also analyzed using this method. The results of the NormFinder analyses are shown in Table 3. Of note, the description of the sample-subgroups had a significant effect on the NormFinder output. However, the NormFinder output with different sample-subgroups and with no subgroups showed two common features: the *ACT* and *PLA2* expression levels had higher stability, whereas the expressions of *RPL13* and *EF1α* were unstable. Additionally, they were always among the least stable reference genes.

While expressing the other sample series, *GAPDH* was the most reliable gene in different plant tissues. However, *RPL13* remained the most variable. In samples of different embryonic development stages, *PLA2* was the most stable gene, whereas *EF1α* was the least stable, with stability values of 0.035 and 0.107, respectively.

### 3.4. BestKeeper Analysis

BestKeeper is a program used to analyze internal reference genes and target gene expression, as well as the inter-gene relationships of the possible reference gene pairs, estimated by performing multiple pairwise correlation analysis using the raw C_t_ values of each gene. Using this software, one can obtain the correlation coefficient (r) and the coefficient of variation (CV) of pairing between each gene, after which internal reference genes with better stability can be identified by comparing the magnitudes of the respective values. The lower the CV value of HKGs, the more stable the expression level of the gene. We ranked the stability of eight candidate genes by comparing CV and r values (Table 4). According to the CV values, the expression levels of *UBQ* and *PLA2* were relatively stable in all samples, embryonic developmental stages, and tissues. However, the expression levels of *EF1α* and *RPL13* were always the most unstable. Our analysis showed that the majority of the eight reference genes had good correlation; *ACT*, and *PLA2*, and *RPL13* had particularly strong intergenic correlations (r > 0.9). The high Pearson’s correlation coefficients indicated that these gene pairs had very similar overall expression patterns. Compared with the previous CV results, *PLA2* and *ACT* were identified as the most reliable standardized reference genes, whereas *EF1α*, *RPL13*, and *UBQ* were unstable and therefore could not be used as reference genes.

### 3.5. Validation of Selected Reference Genes

**Figure 3:**
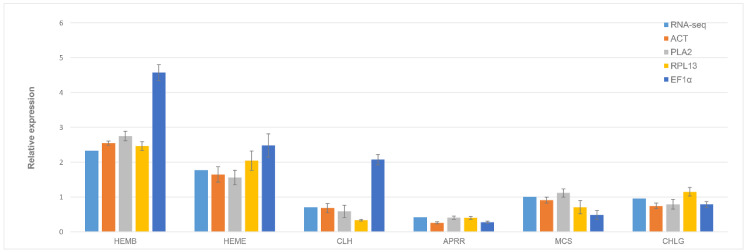
Relative quantification of six genes’ expression between wild type and mutant in *K. paniculata*, as determined by RNA-seq and RT-qPCR with 4 HKGs. Error bars represent standard error of the mean; data shown are means ± SE.

To validate the reliability of the selected reference genes, six interested target gene transcripts (*KpHEMB*, *KpHEME*, *KpCLH*, *KpAPRR*, *KpMCS* and *KpCHLG*) were selected to evaluate the expression level of the mutant sample and the wild type sample by RT-qPCR. The 4 candidate genes (*PLA2*, *ACT*, *RPL13* and *EF1α*) were selected as reference genes, among which *PLA2* and *ACT* were identified as the most reliable standardized reference genes, while *RPL13* and *EF1α* performed poorly and were unstable as reference genes in our analysis. The expression stability varied considerably between the target genes under different reference genes used. The expression levels of the target genes were in general agreement with RNA seq data when we selected *PLA2* and *ACT* as reference genes. However, the target genes’ expression levels varied greatly in the case of *RPL13* and *EF1α*, especially when *EF1α* was used (Figure 3).

## 4. Discussion

*K. paniculata* is an important landscape tree. It is resistant to stress and can adapt to adverse environmental conditions. Additionally, *K. paniculata* has some unique flavonoids with potential medical value. Their synthesis depends on the phenylalanine-initiated flavonoid synthesis pathway. To identify the anabolic metabolism of these unique compounds, the species must be studied at the molecular level. However, previous studies on *K. paniculata* have focused primarily on biological characteristics and pharmaceutical and chemical compositions. At the time of this study, only limited molecular information was available on *K. paniculata* [34,35,36,37]. Therefore, identifying suitable internal reference genes could provide a powerful tool for gene expression analysis in this species. This study was, to the authors’ knowledge, the first to characterize the stability of genes that can be used as internal controls for RT-qPCR studies in *K. paniculata*. The use of internal reference genes could increase the reliability of RT-qPCR data, and the transcriptional level of reference genes should be constant under different experimental conditions.

Reference genes are commonly used to quantify gene expression. Several methods have been proposed to characterize the stability of gene expression and identify the best reference gene in the context of relevant experimental conditions. However, at this time, no consensus has been reached on which method should be used to evaluate the stability of reference gene expression. In *Arabidopsis thaliana*, housekeeping genes, such as those involving basic cellular processes (*EF1α*, *UBQ* and *CYP*) or cell structure maintenance (*ACT*, *TUB*), are generally used as internal reference genes. Under specific experimental conditions, other housekeeping genes have been used as internal reference genes [23]. However, their level of expression is not independent of experimental conditions [32,38], suggesting that the expression stability of any proposed reference gene needs to be tested in advance. We applied three different algorithms to evaluate our references genes. These algorithms (geNorm, NormFinder, BestKeeper) were all Excel-based data operations. In these three operations, the NormFinder and BestKeeper algorithms detected C_t_ values, whereas the geNorm algorithm used relative quantification to estimate the difference in amplification efficiency between PCR reactions. This difference could have affected the determination of stability. Additionally, the BestKeeper algorithm can obtain two different analysis parameters: the CV value and the r^2^ value. By comparing and analyzing the two parameters, it is possible to eliminate the influence of the existing deviation.

The most important observation deriving from the completed analyses provided by the three programs was that each software generated a different set of ranked reference genes—which was not surprising because the three programs were based on different algorithms. Overall, our analyses indicated that *PLA2*, *ACT*, and *GAPDH* were the most reliable internal controls for accurate normalization when evaluating the expression dataset as a whole because these three genes were always classified among the four best-performing reference genes. On the other hand, *EF1α* and *RPL13* ranked poorly in all three software programs, indicating that these two genes were not consistently expressed and should be avoided as internal controls when performing gene expression studies. Selecting more than two reference genes can increase the reliability of the gene expression results, which has been confirmed in soybean [39], peach [40], grape [41], and silkworm [42]. Therefore, when monitoring small changes in gene expression, the use of a single reference gene alone does not always yield accurate quantification results, and a combination of several internal reference genes is required.

Because the genome of *K. paniculata* had not yet been revealed, we identified the gene sequences of eight candidate genes from the *K. paniculata* transcriptome sequencing results and compared these sequences to the *Arabidopsis* database using BLAST. Finally, the primers were designed using the results of the alignment to clone the gene fragments. This method could ensure the efficient cloning of HKGs genes in *K. paniculata* and provide a reference for other species to clone unknown gene sequences. The 18SrRNA gene is commonly used as an internal reference gene; however, because the expression of 18SrRNA is relatively high, the quantitative results are less accurate when the target gene abundance is low [31]. Additionally, the ratio of rRNA to mRNA in the tissues and organs of different physiological states is not constant, and mRNA usually accounts for 5–10% of the total RNA [43]. Although the regulation of rRNA synthesis is independent of mRNA, the transcription of rRNA is susceptible to various biological factors and drugs, and its expression level is not constant. During mitosis, *18SrRNA* significantly reduces or stops expression. Therefore, 18SrRNA was not selected as a candidate gene in this study.

Selection of a suitable and stably-expressed reference gene is dependent on the tissues, cells, and experimental conditions, including the biological or abiotic stress and hormone effects. Therefore, selecting a suitable reference gene is important because it serves as a basis for comparison of the expression changes in the target genes under various experimental conditions. The internal reference genes need to be evaluated and identified under different experimental systems [18,19].

Similar to other species, the expression levels of housekeeping genes are not identical in different tissues or plant developmental stages in *K. paniculata*. Previous studies have analyzed the expression stability of 10 soybean reference genes (*ACT2/7, ACT11, TUA, TUB, EF1α, UBC2, ELF1b, CYP2, G6PD,* and *UBQ10*) in 21 different developmental stages, tissues, and organs [44]. Their results showed that the expression levels of *ELF1b* and *CYP2* were the most stable in all samples, whereas *ACT2/7* and *TUA* were stably expressed only in samples across different developmental stages. Similarly, the expression stability of 10 traditional reference genes (*UBQ11, TUA, 18SrRNA, ACT2, UBQ7, EF1b, TUB, ACT11, EIF4b,* and *CYP*) in eight different developmental stages of poplar was analyzed by [31], who showed that various genes were unstable. The expression of *18S rRNA* was highest and the expression of *TUA* was lowest. Meanwhile, *UBQ11* and *TUA* had the smallest coefficients of variation among different samples, and their expression was most stable, whereas the stability of *CYP* was lowest. Overall, there were relatively stable candidate internal reference genes, even under different experimental conditions and in multiple tissues and organs. Therefore, selection of an appropriate internal reference gene for the specific sample type was an important step for RT-qPCR analysis.

*K. paniculata* is an important ornamental and medicinal plant with features of biotic and abiotic stresses. It is grown widely in China, and has been introduced into the United States, several countries in Europe, Japan, and elsewhere. Currently, this species has only been studied for its biological characteristics and its pharmaceutical and chemical composition. However, there have been no reports, to date, of molecular research on *K. paniculata* in the world. The selection of internal reference genes was the basis for the subsequent study of target genes’ expression patterns. The differentially-expressed target genes in *K. paniculata* populations will be screened through internal reference genes, and a series of molecular markers will be developed further in order to facilitate studies on population diversity and genetic differentiation.

## 5. Conclusions

Our study showed that the expression stability varied among the eight candidate reference genes in different tissues and embryonic developmental stages of *K. paniculata*. Analysis by three programs (BestKeeper, geNorm, and NormFinder) showed that *PLA2* and *ACT* were the best reference genes for RT-qPCR analysis. *GAPDH* and *PLA2* were suitable for different tissues and embryonic developmental stages. However, *RPL13* and *EF1α* were not suitable for use as internal controls because their expression was very unstable. These results were validated in the transcriptome using the expression levels of the interest genes. Our study revealed that multiple reference genes should be used as internal controls to obtain reliable experimental results. Moreover, we screened the internal reference genes and provided a foundation for detailed studies on gene expression in *K. paniculata*.

## Figures and Tables

**Table 1:** Reference genes used for gene expression normalization in *K. paniculate*.

Name	*K. paniculata* Transcriptome ID	Arabidopsis Homolog Locus	Arabidopsis Locus Description	Function
ACT	comp34058_c3_seq1	AT3G12110	ACT11	Encodes an actin that is expressed predominantly during reproductive development
EF1α	comp39196_c0_seq3	AT5G60390	EF1ALPHA	GTP binding Elongation factor Tu family protein
GAPDH	comp35115_c0_seq2	AT1G79530	Glyceraldehyde-3-phosphate dehydrogenase	Encodes one of the chloroplast/plastid localized GAPDH isoforms
UBQ	comp39429_c1_seq8	AT2G47110	UBQ6	polyubiquitin gene the mRNA is cell-to-cell mobile
CYP2	comp31930_c0_seq1	AT2G38730	Cyclophilin	Cyclophilin-like peptidyl-prolyl cis-trans isomerase family protein
RPL13	comp30755_c0_seq1	AT3G07110	60S ribosomal protein L13	Ribosomal protein L13 family protein
PLA2	comp31360_c0_seq4	AT1G06800	PHOSPHOLIPASE A I γ 1	Encodes a lipase that hydrolyzes phosphatidylcholine, glycolipids as well as triacylglycerols
RP II	comp34901_c0_seq1	AT3G22320	RNA polymerase subunit	Non-catalytic subunit common to DNA-dependent RNA polymerases I, II, III and IV

**Table 2:** Reference gene primer sequences and amplicon characteristics.

Name	Forward Primer Sequence [5′–3′]	Reverse Primer Sequence [5′–3′]	Amplicon Size (bp)	Product Tm (°C)	RT-qPCR Efficiency
ACT	ATCAGCAATGCCAGGGAACATA	TCGAGAAGAGCTATGAGTTGCC	227	83.4	1.10
EF1α	AGCCCTCACTATCAGAAACAGC	GTTAAGATGGTTCCGACAAAGC	214	80.6	1.07
GAPDH	AGAGAAACTGACGGGCTATCAA	ATGAAGCTTGTGTCGTGGTATG	209	82.2	0.94
UBQ	ACGGGGTTTTACACTATGAACG	TCGGATAACCTCTTCCAACAGT	214	80.6	0.90
CYP2	TCGAAGAATACGATTGGGTTTT	TCAACGAATCCGTTACAAACAC	193	83.8	0.79
RPL13	GACCCTCTAGGGAACGATTCTT	CTCGTCAGAAGAAAGCTGTGAA	216	83.0	1.00
PLA2	AAATTAACGAGGACACCAATGC	GGGTATGGATATGGCGATCTTA	212	79.0	0.95
RP II	CAACTGTGTTTCCTTCACCGTA	TGGTGGTTCAACAGAATTTGAC	200	81.2	1.07

**Table 3:** Ranking of candidate reference genes in order of their expression stability as calculated by NormFinder.

Ranking Order	All Samples						Plant Tissues		Embryo Developmental Stages	
	No Subgroups		2 Subgroups		6 Subgroups					
	Gene	Stab.	Gene	Stab.	Gene	Stab.	Gene	Stab.	Gene	Stab.
1	ACT	0.023	PLA2	0.015	PLA2	0.040	GAPDH	0.018	PLA2	0.035
2	PLA2	0.026	GAPDH	0.019	ACT	0.040	ACT	0.032	ACT	0.042
3	GAPDH	0.035	ACT	0.022	GAPDH	0.042	PLA2	0.044	CYP2	0.056
4	RP II	0.062	UBQ	0.039	RP II	0.068	RP II	0.058	GAPDH	0.062
5	CYP2	0.082	RP II	0.040	UBQ	0.083	EF1α	0.060	RP II	0.068
6	UBQ	0.085	EF1α	0.046	CYP2	0.083	CYP2	0.078	UBQ	0.069
7	EF1α	0.104	CYP2	0.052	EF1α	0.089	UBQ	0.097	RPL13	0.081
8	RPL13	0.151	RPL13	0.063	RPL13	0.122	RPL13	0.162	EF1α	0.107

**Table 4:** Ranking of the eight genes according to correlations between reference genes and BestKeeper index.

Ranking Order	All Samples		Embryo Developmental Stages		Plant Tissues	
	CV	r	CV	r	CV	r
1	UBQ	ACT	CYP2	PLA2	UBQ	ACT
2	PLA2	RPL13	UBQ	RPL13	RP II	GAPDH
3	CYP2	PLA2	PLA2	CYP2	PLA2	RPL13
4	RP II	GAPDH	ACT	ACT	CYP2	EF1α
5	ACT	RP II	RP II	GAPDH	GAPDH	PLA2
6	GAPDH	EF1α	GAPDH	RP II	ACT	RP II
7	EF1α	CYP2	EF1α	EF1α	EF1α	CYP2
8	RPL13	UBQ	RPL13	UBQ	RPL13	UBQ

## Data Availability

The data presented in this study are available on request from the corresponding author.

## Supplementary Materials

The following supporting information can be downloaded at: https://www.mdpi.com/article/10.3390/genes13050714/s1, Figure S1: Melt curves of the eight candidate reference genes; Figure S2: Electrophoresis gel of the eight candidate reference genes; Table S1: The sequences and primers of the genes selected for validation.
