# Supplementary material for: Identification and Validation of Reliable Reference Genes for Gene Expression Studies in Koelreuteria paniculata
Source: Genes (Basel). 2022 Apr 19;13(5):714. doi: 10.3390/genes13050714 (PMC9141280; doi:10.3390/genes13050714)
Supplement: Supplementary file 1 [file genes-13-00714-s001.zip › genes-1686645-supplementary.pdf]

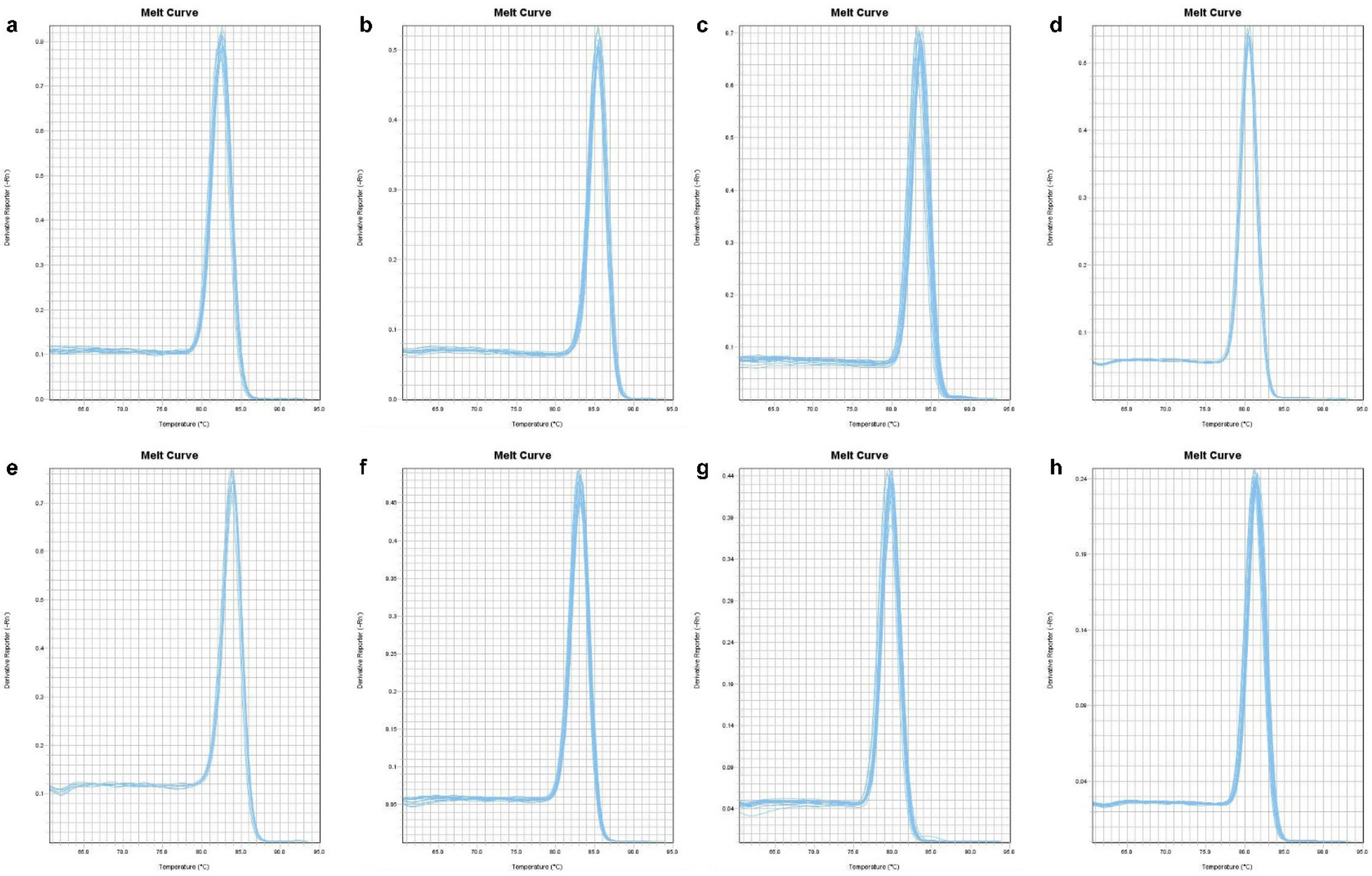

Supplement Figure S1 Melt curves of the eight candidate reference genes

Note: X-axis: Temperature (°C); Y-axis: Derivative Reporter (-Rn ‘); a: ACT; b: EF1α; c: GAPDH; d: UBQ; e: CYP2; f: RPL13; g: PLA2; h: RP II.

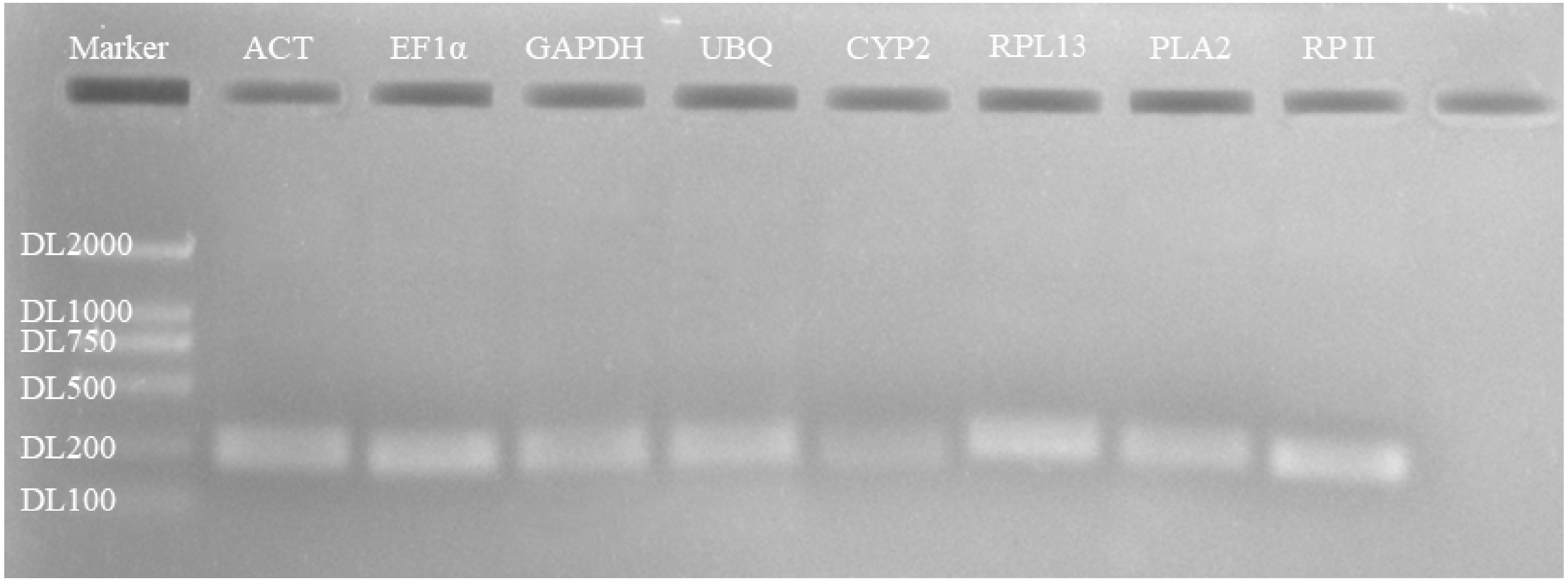

Supplement Figure S2 Electrophoresis gel of the eight candidate reference genes

Supplemental Table S1:

qRT-PCR primers are capitalized and underlined.

KpCHLG

ATGTCGTCGGCTGCGCTGAACACAGTTTTGTCCACTAGAATATCCAACGTCGTCAACGCCAACAACCGCCGAGGCCGAGTTGGAGTTCGAACTCACCC
CGTACTTAACCCGGCTTCCGTTTCCTTCACCGCGCGGAGATTGACTGTGAGAGCTCAGGAGACCGATGCAAAAGAAGCTAAATCTCAGGCGCCTGACA
AAGCGCCGGCTGCAAATGGGTCGAGTTTCAATCAGCTTCTAGGCATCAAAGGAGCTGCACAAGAAACTAATAAATGGAAGATTCGTCTTCAACTAACG
AAGCCTGTTACTTGGCCTCCATTGGTTTGGGGAGTAGTTTGTGGAG**CCGCTGCTTCTGGAAACTTT**CACTGGAATTTGGAAGATGTTGCCAAATCAAT
TGTTTGCATGATGATGTCTG**GCCCCTGTCTCACTGGTTAT**ACACAGACCATGAATGATTGGTACGATCGAGAGATTGATGCTATTAATGAACCTTACCG
TCCCATTCCTTCAGGAGCAATATCTGAGAATGAGGTCATCACCCAAATCTGGGTTCTGCTTCTAGCAGGCCTTGGGTTAGCTGGTATATTAGATGTTTGG
GCCGGGCATAACTTTCCTATAGTGTTTTACCTAGCTCTGGGTGGATCCTTGCTTTCGTATATCTACTCTGCTCCACCTTTAAAGCTCAAACAAAATGGATG
GATCGGAAATTTCGCTCTGGGAGCAAGCTATATCAGTTTGCCATGGTGGGCTGGCCAAGCTTTGTTTGGAACGCTTACACCTGACATAATAGTTCTCAC
GCTCTTGTACAGCATAGCAGGGTTAGGTATTGCCATCGTGAATGACTTTAAGAGTATCGAAGGAGATAGAGCACTGGGACTGCAGTCTCTTCCAGTTGC
TTTTGGTGCTGAAGCTGCTAAATGGATTTGTGTTGGCGCCATCGACGTAACTCAATTGTCAGTGGCTGGTTACCTACTTGGGGCTGGTAAACCATTATAT
GCCCTAGCTCTGCTTGGCTTGATTATTCCCCAAGTTGTCTTCCAGTTTAAATACTTCCTCAAAGACCCTGTAAAATATGATGTAAAATATCAGGCTAGTGC
CCAGCCATTTCTTGTGCTCGGTCTACTTGTGACTGCTTTGGCAACAAGCCATTGALEFT

KpAPRR

ATGGGAGAGTTGGTGTTGAGTAGTGAAGAAATGGAAGTTGTGAAGAACACGACAACAACAACAGCAGCAGCAGCAGCAGAAGCAGAGGAAAAGAA
AGACGGGTTGGTGAAATGGGAGAGGTTCTTGCCAAGGATGACTTTGAGGGTTTTGCTTGTTGAGGCTGATGATTCTACCAGACAAATTATATCTGCCCT
TTTAAGGAAGTCCAGCTACAGTGTTAATGCCGTTCCTGATGGCTTGAAGGCTTGGGAGATTCTGAAGGGAAGACCAGAAAACATAGATATCATATTGAC
AGAAGTTGATTTACCTTCGATATCTGGATTCGCGCTTCTTACGCTGATTATGGAGCATGAGATTTGCAAAAACATCCCTGTTATAATGATGTCCTCACAGG
ATTCGGTTAGCACTGTATATAAATGCATGTTGAGAGGTGCTGCTGATTATCTTGTTAAGCCTGTAAGGAGGAACGAGTTGAGAAATCTGTGGCAGCATG
TATGGAGAAGACAATCTACAACCATGGGTGGAAATGTTGCCCAAGATGAGAGTGTTGGACAGAAGAGGATTGACGCCTCTTCTGAAAATGCTGCTGCA
AGTAATCATTCTAGTGGTTACATGGCTTGTATCCAGAGAGATGAAGAGTGCAACGAGAAAGGGAGTGATGCTCAGAGCTCTTGTACAAAACCGGACTT

CGAAGCTGAGAGTACACATGTGGAAGTTACGCAGGATATGTCAACACAGTTAGGGACTAAATGCTTACCGGATGATGTAAAGATGCAGAAGCATAAAG
GATGTGTTAATTACGTCCAGAAATCACCTGTGCATGAGACTGAAGCAGGAGGATCAGGAGTGGCTGTTGGTGAGGATGCTAACCGAGAAAAACAGAA
TTTGGGCAAGGATGTTGATCCAGATAGTCAAAGGAAGGATCTTATTATCACCAGTGAGGCCAGTAATGCCCATGTCAATTCTTCCAGAGAAGTCATCGA
CTTCATGGGAGCCTTGAACAATCTCAATACTTCTTCAAACAATTGCACTAGCAAGTTTGATTCTTCTTCACAATTGAACTTTTCCTTGCGAAGATCTCAT
CCTAGTGGCTTCGAGAATCAAGTTGCTCAAAAGACATTTACCCTCAAGCATTCTAATGCCTCAGCCTTTACACGGTATACCAACAAGCCAGTGCAGTCT
CTCCATTCATCTTTTGCAAGTGCTTGCAATCAGCAGAAAGAATTTGATCCTGAGAAGAATTTGTCCAATATTGTTACCACTGGTAACTC**CGATACTCCT**
GTTGCCATGCTAAGTACTCAAAGAAGTATCAATTCTCTGGCTACTAGCCAATTTAAACAATCTGAGGTTGAAACTTCATGCCCTCGACAAAGAGTACG
CCCTGTACAAGTTCC**AGTTGAAGGTGTGAGGCTGA**ATAATGCCTGCACCGGCTATGGCTCTGTACTCCCTAATATATTTTGTGCACAATCGGGTCTATC
ACCGAGTCCAAATCCAAGTCCAGCCAATCAACAGGAACTTACCTTTCGAGTTAATCAAATGTATCACTCCAATGTAGAAAATGGTAATTGCGGACAGTT
ATATGATCGACTTGGTGAAAGTGCAATCAATTCCACCAACCAAGCTATTCACAGGTTGGATTCTCTGGAGGATCGTGGACAGATTTCTCCCACCACTGA
TCAGAGTGCAAGTAGCAGTTTTTGTAATGGTGCTATAAGTCGTCTTAACAGCATGGGTTATGGAAGCACTTGTGGAAGCAACAACAATGTTGATCAAGG
TGCTGTTGGCAGGACTGCTGCTGAGAGTAAAAATGAAGAAGGTTTCATACCTTCCTGTGGAAACTCACATCGATCAATCCAAAGAGAAGCGGCTCTAA
ATAAGTTCCGCTTGAAGCGGAAGGATAGGTGCTACGAGAAGAAGGTTCGGTACGAGAGTAGAAAAAAAACTTGCAGAACAGCGCCCCAGAGTGAAA
GGACAATTTGTTCGTCAAGTGCAGACTGA

KpHEME

ATGAGCTTCTC**TTCTTTGACAAGTGCGTGCA**GTTTCTTAGGAAGCTCGTCGGTGCAGTTAGGTTTCCACTGTGATTCTCTAAAGGGGGTTCTGTTTTC
TTCTTCCAAGAGTCAAAAATCTACTCTAAAAAAAATCACTG**TTTCCTGCTGCTCCTCTTCA**TCTGATCCACTACTGGTTAAGGCTGCAAAAGGCTATC
CTGTAAGTCGGCCACCAGCATGGATGATGCGTCAGGCAGGAAGGTATATGGCTGTTTACCGAAAGCTTGCAGAAAAGTACCCATCCTTCAGGGAGAGG
TCAGAGACAACTGATCTCATTGTGCAAATTTCTTTGCAGCCTTGGAAGCTTTTCGTCCTGATGGAGTTATCATTTTTTCTGACATACTTACACCACTACCC
GCATTTGGTGTCCCTTTTGACATAG

KpCLH

GGAACAAAATATCAGGGGCATGACAATATGTCTTCTACTTCTTCTGCAACTACAAGTGTTTTTGAGACCGGAAAATACACAACCACGCTTCTAAGGGTG
GAACCATCTGGGACGACGACGACATGCGTCTCAATTGGTTCATCAGTGGATGTTCCACTTCCACCACCAAAGCCACTTCTGATTGCCACCCCTAGTGA
AGCCGGAGAGTTCCCACTTCTGCTTTTCCTCCACGGTTACCTTCTTTATAACTCCTTCTACTCTCAGCTACTCCAACATGTTGCTTCTCATGGTTTCATCG

TCATTTCTCCTCAGTTATATGAGGTGGCTGGAGCAGACTCAACTGGTGAGATTAAATCTGC**AGCTGCAACGACAAATTGGT**TATCTGAAGGACTCCGT
GACTTGCTTCCACCAAATGTTCACCCAAATCTAAGTAAGTTAGGTCT**TGCTGGTCATAGTCGTGGAG**GCAAGACTGCCTTTGCACTGGCTCTAACAA
AAGCAGCCACTACTTTGAAAATTTCAGCCTTAATAGGCATAGACCCGGTTGATGGTATGGATAAAGGGAAACAAACTCCACCACCAGTACTGACTTAC
GTTCCTCATTCATTTGATCTTGATATGGCGGTGATGGTGATCGGTTCAGCCTTAGGTGAAGTAAAAAGAAACCCTCTATTCCCTCCTTGTGCTCCAAAGG
GAGTTAATCATGAAGACTTCTTCAACGAATGTCGAAAACCAGCCTGTCATTTTGTGGCTAAGGACTATGGTCATCTCGATATGTTGAATGATGATACAAA
AGGGATTAGAGGGAAAACTACTTATTGTCTCTGTAAGAATGGCAAGTCCAGGGAACCCATGAGGAGGTTTGTTGGAGGTATTGTTGTTGCTTTTATGAG
AGCTTATTTGGATGGTGATACAAGTGAATTGATGTCTATAAGATTTGGGCATGAAACTGCACCTGTGGAGCTTAAAACTGTTGAATTTCTTGAGTGA

KpHEMB

ATGATGAGACGGGTGCATCAATTATGTAAACAAGCTGTTTCCCAGGCCCGAGCTGG**AGCAGATGTTGTGAGTCCCA**GTGATATGATGGATGGTCGTGT
AGGAGCAATTCGAGCAGCTCTTGATGCAGAAGGCTTTCAGCATGTTTCTATCATGTCCTATACAGCGAAGTATGCAAGTTCATTTT**ATGGTCCATTTCG**
AGAGGCACTAGATTCCAATCCACGTTTTGGAGACAAAAAAACTTATCAGATGAATCCAGCAAATTACAGAGAGGCCCTTGTCGAGGCCCGTGAAGAT
GAATCTGAAGGAGCTGACATCCTTTTGGTGAAACCAGGTCTTCCTTATTTGGATATTATAAGACTACTCAGAGATAAGTCTCCTTTGCCAATTGCAGCAT
ATCAGGTTTCGGGTGAATACTCAATGATCAAGGCTGGTGGGGTTCTCAAGATGATTGGACGAAGAGAGGGTTATGATGGAATCATTAATGTGCCTCCGG
CGGGCTGGTGCTGA

KpMCS

ACAGGACTCTCTCTTCCAGCAGCGAGCTTCCCGGACTCGGTTCAATTTCTGATCCAGTTTTCAAACTTTGAAGCGAGTTATGGTGTTCTTCAGGAGCCC
TCACTGCGCTTTCTCGTCTACGTTCTCGTGTTAGCCAACAATCTGGTCTCTCCAATTTCTGTCAGATGGCTTCAGATGCAAAGCTCTTCTTCTGATCTTG
ACCTTCATTCTCAGCTCAAGGAGTTGATTCCAGAACAACAGGAGCGCCTGAAGAAAGTCAAGTCAGAATATGGGAAGTCCCAGTTGGGAAATATTACT
GTTGATATGGTAAT**TGGTGGCATGAGAGGAATGA**CTGGCCTACTGTGGGAAGGCTCTTTACTTGATCCTGATGAGGGAATTCGATT**TAGGGGCTTGT**
CGATTCCTGAATGCCAACAAGTATTACCCAGCTGCAAAGCCTGA
